# The Interactions between Ciprofloxacin and Parenteral Nutrition Admixtures

**DOI:** 10.3390/pharmaceutics12010027

**Published:** 2019-12-27

**Authors:** Aleksandra Gostyńska, Maciej Stawny, Katarzyna Dettlaff, Anna Jelińska

**Affiliations:** Department of Pharmaceutical Chemistry, Poznan University of Medical Sciences, 6 Grunwaldzka, 60-780 Poznań, Poland; gostynska.aleksandra@spsk2.pl (A.G.); dettlaff@ump.edu.pl (K.D.); ajelinsk@ump.edu.pl (A.J.)

**Keywords:** ciprofloxacin, parenteral nutrition, zeta potential, compatibility, drug interactions

## Abstract

Background: Co-infusion of parenteral nutrition (PN) and other drugs increases the risk of the interaction between drug and PN admixtures that can cause embolization of small blood vessels, resulting in potentially fatal consequences, including pulmonary embolism, or liver and retina vascular damage. The present study aimed to determine the compatibility between ciprofloxacin (CF) and eighteen compounded PN admixtures in order to assess the possibility of their co-administration via Y-sites. Methods: CF and PN admixtures were mixed at two volume ratios (1:1 and 2:1) and potential interactions were examined using visual inspection, and measurements of pH, osmolality particle size, and zeta potential. The analyses were conducted immediately after sample preparation and after four hours of storage. Results: The compatibility tests showed that the addition of the CF to the PN admixtures did not cause any color change or sign of destabilization in the fat emulsion. However, precipitation was observed in formulations containing higher CF concentrations and, in the case of lower CF concentrations, in formulations containing magnesium and calcium ions at a molar ratio of 2:1. Conclusions: The administration of CF and PN admixtures via the Y-site should be avoided or performed only with PN admixtures for which compatibility has been confirmed and the CF concentration in samples is 1 mg/mL at a molar ratio of magnesium to calcium ions of 1:1.

## 1. Introduction

The treatment of bacterial infections is a challenge to modern medicine, as more than 50 percent of patients in intensive care units suffer from bacterial infections [1]. The mortality rate of infected patients is twice as high as for uninfected patients, and antibacterial treatment accounts for up to 40 percent of the cost of hospitalization of critically ill patients [1].

Ciprofloxacin (CF) is a broad-spectrum chemotherapeutic agent characterized by significant potency against Gram-negative bacteria and atypical pathogens. CF is often used to treat moderate to severe bacterial infections, especially those of the respiratory and urinary tracts in critically ill patients [2]. However, the overuse of CF has caused an increase in the drug resistance of Gram-negative bacteria, particularly *Pseudomonas aeruginosa*, *Klebsiella pneumonia*, *Escherichia coli*, and *Acinetobacter* spp. [2].

Critically ill patients often require nutritional support in the form of enteral or parenteral nutrition. Parenteral nutrition (PN) involves the intravenous supply of all necessary nutrients and frequently coexists with the infusion of other parenteral drugs. In patients with limited access to blood vessels, it is necessary to interrupt PN infusion or combine two pharmaceutical preparations (PN admixture and another parenteral drug) into one infusion using the Y-site. Such a procedure raises the risk of interaction in the pharmaceutical phase and may lead to precipitation or destabilization of the fat emulsion. Administration of two incompatible preparations in one infusion may endanger the patient’s health and life, resulting in the economic consequences of treating medical complications. Therefore, the determination of principles for administration of drugs and PN admixtures via the Y-site is a vital aspect of clinical practice, especially in light of the reports of Taxis and Barber [3], who have shown that about a half of all intravenous preparations and infusions are administered incorrectly. Administration of incompatible preparations causes approximately 20 percent of all medical errors and constitutes almost 89% of errors in drug administration [4,5].

In one of the first studies on the supply of drugs with PN admixture via the Y-site, the volume ratio of both preparations during infusion was determined. The drug-PN admixture ratio obtained at that time was 1:1 (*v*/*v*), to be reproduced in subsequent studies on parenteral drug compatibility [6]. However, when two simultaneous infusions are administered, e.g., a 12–24 h PN infusion and a 15–60 min infusion of another intravenous drug, this proportion may differ significantly. In this case, the volume ratio of the drug to the PN admixture should be calculated considering the infusion rate of both preparations [7] or an appropriate drug-PN admixture combination should be obtained, simulating the supply of both preparations by using an infusion pump [8].

PN admixtures are the most complex drugs used in modern medicine. Up to 50 different substances are mixed together in one package, which raises the possibility of physicochemical interactions [9,10]. Administering a PN admixture simultaneously with another infusion drug or adding a drug to the PN admixture carries a high risk of physical or chemical incompatibility.

In the case of supply using the Y-site, chemical incompatibilities are considered less significant due to the short time that both preparations are in contact (1–2 min) [11]. However, when drugs are added to the PN admixture, any resulting chemical incompatibility can significantly affect the treatment outcome. Then it is necessary to confirm the chemical stability of the added substance. A decrease in the drug content by more than 10% during preparation, storage, and administration disqualifies its addition to the PN admixture [12]. Regardless of the manner in which the PN admixture and the drug are supplied (simultaneous supply via the Y-site or adding the drug to the PN admixture), physical incompatibilities may occur manifested as precipitation, lipid emulsion destabilization, or color change [9,10]. The formation of lipid emulsion droplets larger than 500 nm in diameter or precipitation of insoluble sediment can cause embolization of small blood vessels, leading to potentially fatal effects, such as pulmonary embolism or capillary damage of the liver or the eye retina [13,14,15,16,17]. The risk of physical incompatibility is associated with pH differences between preparations, the concentration of the drug added and PN admixture ingredients, the temperature and light exposure during storage and administration. PN admixture is an oil-in-water emulsion with a pH of 5.5–7.5, stabilized by egg phospholipids [18]. Lowering the pH below 5.5 may lead to destabilization of the emulsion as a result of a change in charge on the micelle surface and, consequently, phase separation of the system. High electrolyte concentrations, especially divalent and trivalent cations, may also affect the surface charge of lipid droplets destabilizing the oil-in-water system [19].

The aim of the study was to determine interaction between CF and PN admixtures when infused simultaneously using the Y-site. In order to find the relationship between the composition of PN admixture and CF compatibility, 18 different PN admixture formulations were used in the study, which differed in the lipid emulsion type and the content and molar ratio of electrolytes. The CF and the PN admixtures were mixing together at two volume ratios 1:1 and 1:2, which corresponded to CF concentrations of 1.33 and 1.00 mg/mL, respectively. In order to determine possible interactions, measurements were carried out of pH, osmolality, zeta potential, and mean droplet diameter (MDD) of lipid emulsion.

## 2. Materials and Methods

### 2.1. Composition of PN Admixtures

Eighteen formulations of PN admixtures were selected for the study differing in the type of lipid source and the content of electrolytes (Table 1). The composition of the PN admixtures was based on literature recommendations for adult hospitalized patients. Two different types of lipid emulsion were used. The PN admixtures containing Lipofundin^®^ MCT/LCT (PN1-PN13) (B. Braun Melsungen AG, Melsungen, Germany) consisted of long-chain triglycerides (LCT) and medium-chain triglycerides (MCT). The PN admixtures containing Smoflipid (PN14-PN18) (Fresenius Kabi AB, Upsala, Sweden) were based on a lipid emulsion consisting of MCT, LCT, olive oil), and triglycerides of omega-3 acids.

### 2.2. Preparation of PN Admixtures and CF–PN Samples

The PN admixtures were prepared in aseptically under a laminar-flow air hood, using a Pinnacle B. Braun automatic compounder (B. Braun Melsungen AG, Melsungen, Germany) ensuring high-precision dosing. The final volume of 1800 mL was packaged in an ethylene-vinyl acetate (EVA) bag (B. Braun Melsungen AG, Melsungen, Germany).

The samples were prepared by mixing the PN admixtures with a CF solution for infusion (Ciprofloxacin Kabi 200 mg/100 mL, Polpharma S.A., Starograd Gdański, Poland) at ratios based on infusion rates for both medications. The infusion time for the CF (0.5 h) was specified in the summary of product characteristics. The infusion time for the PN admixtures (20 h) was based on clinical practice. The infusion rates were calculated on the basis of the infusion times and the drug volumes (100 and 1800 mL for the CF and the PN admixtures, respectively). The volume ratios of the CF and the PN admixtures in the infusion line (1:1; 1:2) were obtained by dividing the drug infusion rate by the infusion rate of the PN admixture. The concentrations of the CF in the CF–PN samples at the volume ratios 1:1 and 1:2 were 1.33 mg/mL and 1.00 mg/mL, respectively. Each sample was examined immediately after preparation and after four hours of storage at 25 ± 1 °C. We prepared three independent samples for each combination of CF–PN admixtures in each volume ratios.

### 2.3. Visual Inspection

In accordance with the European Pharmacopoeia [20], all PN admixtures were visually assessed for the presence of visible particles and/or color change. Visual inspection was performed against a black-and-white contrast background by two observers. Following the pharmacopeial requirements for intravenous lipid emulsions, to consider PN admixtures as compatible with CF, they must be practically free from visible particles, and no precipitation can be detected by either observer upon visual inspection.

### 2.4. pH Evaluation

The pH was measured at room temperature, using Mettler Toledo Seven Compact pH/ion S220^®^ pH-meter (Mettler Toledo, Columbus, OH, USA). Each sample was measured in triplicate, and the results were expressed as average ± standard deviation.

### 2.5. Determination of MDD of Lipid Emulsion and Zeta Potential

The MDD of lipid emulsion and zeta potential (*ξ*) of PN admixtures were measured at 25 °C, using a Zetasizer Nano ZS (Malvern Instruments, Malvern, UK) by dynamic light scattering (DLS) and laser Doppler velocimetry respectively. The sample preparation, particles size, and zeta potential determination were performed according to the methodology described in our previous work [21]. The results of droplet diameter measurements were presented as follows: MDD (intensity weighted mean droplet diameter). All the measurements were performed in triplicate, and the results were expressed as average ± standard deviation.

To consider PN admixtures compatible with CF, the following criteria must be met: the size of lipid droplets expressed as intensity-weighted MDD cannot exceed the pharmacopeial limit of 500 nm. This criterion was set for the US pharmacopeia method I for the determination of the mean droplet size of lipid injectable emulsions [22].

### 2.6. Osmolality Determination

The osmolality was measured at room temperature, using 800 CLG TridentMed^®^ osmometer (TridentMed s.c., Warsaw, Poland). 100 μL of CF, PN, and CF–PN admixtures was injected into the Eppendorf tube, and the tube was placed in a cooling chamber. The measurements were performed immediately (time = 0 h) after samples preparation and 4 h later. Each sample was measured in triplicate, and the results were expressed as average ± standard deviation.

### 2.7. Fourier Transform Infrared Spectroscopy (FT-IR)

The precipitate was filtered and dried. Then, 1 mg of precipitate (tested sample) was micronized and mixed with 300 mg of KBr to produce tablets (1.3 cm × 0.1 cm). The reference spectrum was obtained by using the same tableting method by mixing 1 mg of CF (Sigma Aldrich, Saint Louis, MO, USA) with 300 mg of KBr. FT-IR spectra were collected on an IRAffinity-IS Fourier Transform Infrared Spectrophotometer (Shimadzu, Kyoto, Japan) instrument in the range of 400–4000 cm^−1,^ with a resolution of 4.0 cm^−1^ and 40 scans. The identity of CF was confirmed by comparing the spectrum of precipitate with the spectrum of the reference sample and by calculating the purity factor. Purity (*P*) is a factor that indicates the degree of matching between two spectra. This factor is given by the least-squares-fit coefficient that is calculated for every intensity pair of the two spectra being compared. *P* factor can be calculated by using the following formula:

$$P=\frac{\sum_{i}^{n}\left(s_{i}-\bar{s}\right)\times (r_{i}-\bar{r})}{\sqrt{\sum_{i}^{n}{\left(s_{i}-\bar{s}\right)}^{2}\times \sum_{i}^{n}{\left(r-\bar{r}\right)}^{2}}},$$

where *s_i_* and *r_i_* are the respective intensities for the same horizontal coordinate value, and *n* is the number of data points; $\bar{s}$ and $\bar{r}$ are the average intensities of each spectrum.

The purity value is between 0 and 1, where 0 indicates no identity between the two spectra, and 1 indicates that the two spectra are identical.

### 2.8. Statistical Analysis

The data were analyzed by using Statistica 12 software (StatSoft). Two-way analyses of variance (ANOVAs) was used to determine the statistical significance between samples. The a priori level of significance was *p* < 0.05. In the case of a major effect or interaction, significant differences between the tested TPN admixtures (with drugs) and the reference samples under the study conditions were identified using Tukey’s HSD post hoc tests.

## 3. Results

An ANOVA analysis of the main effects for the CF and the PN admixtures showed a statistically significant (*p* < 0.05) effect of the CF and PN admixture formulations on the pH, osmolality, zeta potential, and MDD of the lipid emulsion. A statistically significant (*p* < 0.05) effect of storage time on all parameters was also observed, with the exception of osmolality.

The pH of the PN admixtures (PN1–PN18) ranged from 6.34 to 6.72 (Table 2). The addition of the CF caused a significant decrease in the pH of all formulations. The pH of CF-(PN1–PN18) admixtures ranged from 5.90 to 6.33. The osmolality of all PN admixtures ranged from 848 ± 1 mOsm/ kg of H_2_O to 1281 ± 3 mOsm/kg of H_2_O and decreased significantly (*p* < 0.05) after adding the CF and ranged from 441 ± 1 mOsm/kg of H_2_O to 730 ± 5 mOsm/kg of H_2_O (Table 3).

The zeta potential of the PN admixtures was between −22.47 ± 0.81 mV and −8.38 ± 0.15 mV (Table 2). The lowest values were observed for PN18, and the highest for PN4. The highest zeta potential values for samples with the CF were observed for CF–PN4 at the ratio 2:1 (*v*/*v*). The lowest zeta potential values were observed for CF–PN18 at a 1:1 (*v*/*v*) ratio (Table 3).

The MDD of PN1–PN18 ranged from 216.5 ± 2.4 nm to 338.8 ± 3.4 nm (Table 2). The addition of the CF caused a slight shift of this range toward lower values (from 212.5 ± 2.2 nm to 329.0 ± 10.6 nm). The addition of the CF to the PN admixtures decreased the MDD for all samples except CF–PN11 and CF–PN17, at the ratio 1:1 (*v*/*v*), for which no change or a slight increase in the MDD was observed (Table 3).

All PN admixtures were evaluated visually (against white and black backgrounds) upon preparation, after the CF addition and four hours later. The PN admixtures without any CF content were white homogeneous oil-in-water emulsions and showed no signs of reversible (sedimentation, creaming) or irreversible (coalescence and flocculation) degradation of the lipid emulsion during four hours of storage. The addition of the CF to the PN admixtures did not cause any color change or visible degradation of the lipid emulsion immediately after the addition, whereas, after four hours of storage, a sedimenting flocculent precipitate was observed in the CF-(PN5–PN8) and CF-(PN17–PN18) at the ratio 1:1 (*v*/*v*) and in all CF–PN at the ratio 2:1 (*v*/*v*).

**Figure 1:**
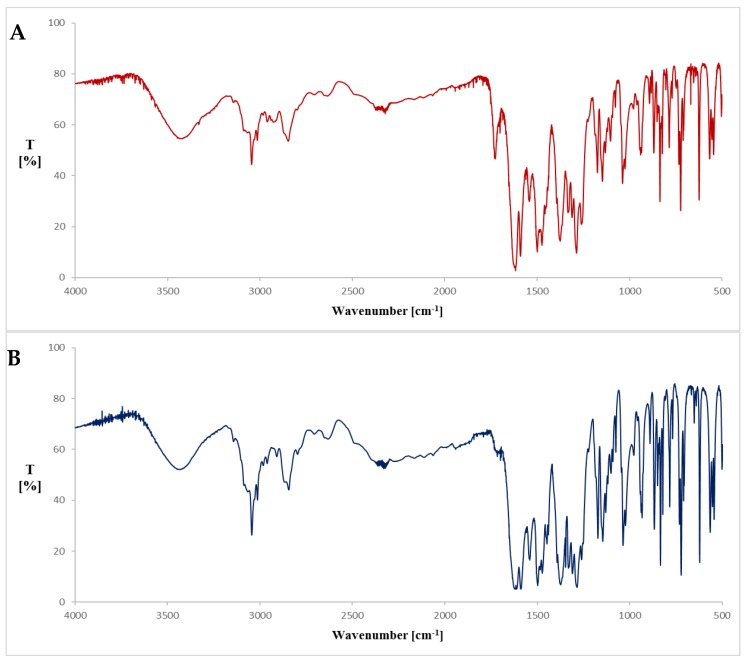
FT-IR spectra of precipitate (**A**) and reference sample of CF (**B**).

**Figure 2:**
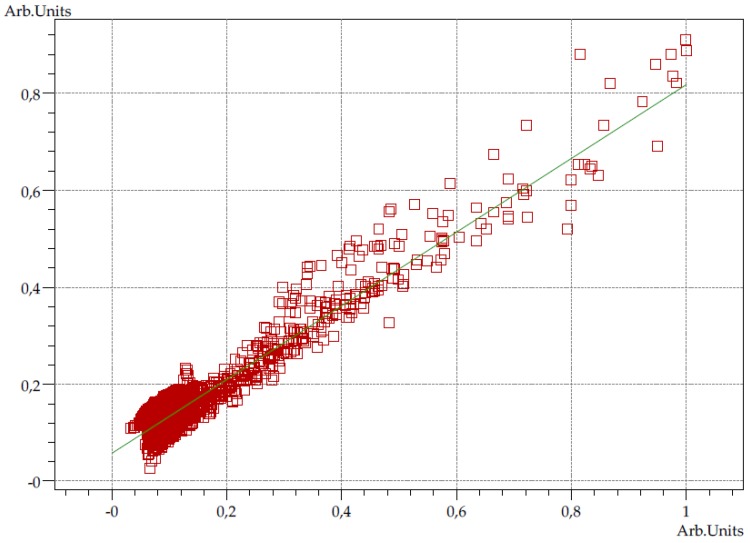
Correlation curve of the intensities of the precipitate spectrum and the reference spectrum.

The precipitate was dried, and spectra were plotted, using the KBr tablets method. The spectra of precipitate and reference sample of CF are presented on Figure 1. The calculated purity factor was 0.9552, indicating high spectral matching. The purity graph shows the correlation curve of the intensities of the source spectrum with respect to those of the reference spectrum (Figure 2).

Table 4 shows characteristic bands of the precipitate and the reference samples. The stretching vibration at 3436.21 cm^−1^ is attributed to the OH in the carboxylic group. The absorption band at 1621.67 cm^−1^ is associated with the symmetric stretching of C=O of pyridone moieties. Stretching band assigned to the C=C and C–N are seen at 1587.91 cm^−1^ and 1284.60 cm^−1^, respectively. The absorption band at 1036.26 cm^−1^ is due to the C–F stretching vibration, while the vibration at 722.34 cm^−1^ indicates the absorption peak of secondary amine (–NH).

## 4. Discussion

The PN admixtures to be studied were prepared at a volume of 1800 mL each, with a glucose content of 450 mL. The PN1–PN12 admixtures contained Lipofundin MCT/LCT 20%. The PN13–PN18 admixtures differed from PN1–PN6 only in the lipid emulsion used (Smoflipid 20%). Formulations PN1–PN8 and PN13–PN18 were characterized by a high content of monopositive ions in comparison with a low content of such cations in formulations PN9–PN12. The PN1–PN4 and PN9–PN16 formulations contained a greater amount of divalent cations (magnesium and calcium). In addition, the molar ratio of formulations with a higher content of divalent cations was 1:1, and those with a lower content of magnesium and calcium ions had a molar ratio of 2:1.

The sedimentation observed in all samples containing the CF at a volume ratio of 2:1 probably resulted from a high CF concentration in the samples (1.33 mg/mL) and the acid–base balance manifested by the pH of the CF–PN admixtures of about 6, with a possible effect on CF solubility. According to the Henderson–Hasselbalch equation, when the pH of the solution exceeds the pKa of the functional group that can be protonated, the deprotonated form dominates. Thus, as the pH increases, the basic functional groups become non-ionized and less soluble in water, while acidic functional groups become ionized and more soluble in water [12]. CF has both a carboxyl group (pKa ≈ 6.1), as well as the piperazine basic group (pKa ≈ 8.7), which causes that this drug has a pH-dependent solubility. Depending on the pH, the CF can occur in the following ionic forms: cationic at acidic pH, zwitterionic at pH values comprising pKa, and an anionic at basic pH [23]. CF is poorly water-soluble drug and exhibits a U-shaped pH-solubility profile, with high solubility at pH values below 5 and above 10, and minimum solubility near the isoelectric point, which is close to neutral pH [24]. At pH 7.4, the majority of CF molecules exist in the form of zwitterions with a global neutral charge, and it is the least soluble. In contrast, at pH 3 the solubility of CF increases significantly. At pH 5, about 90% of CF is transformed into a cationic form with a greater solubility than the zwitterion form, and its solubility increases with the pH lowering [25]. At the pH characteristic for mixtures of CF and PN admixtures (5.82–6.33), we predicted that the solubility of CF would still be low.

In the case of CF–PN admixtures at a 1:1 volume ratio (CF concentration = 1 mg/mL), the presence or absence of precipitation was probably also caused by the influence of cations present in the PN admixture formulations: monovalent (sodium and potassium) and divalent (calcium and magnesium) ions. In the presence of metal cations (except magnesium ions), CF in an acidic environment forms well-soluble complexes, while, in the presence of magnesium sulfate, the solubility of CF slightly rises as the amount of magnesium sulfate increases, followed by a gradual decrease. At higher concentrations of magnesium sulfate, precipitation has been observed [26]. Turel et al. [27] showed that CF forms adducts with magnesium sulfate, in which magnesium is not bound to the drug molecule but is coordinated by six water molecules to form aquacation [Mg(H_2_O)_6_]^2+^. In our study, for the CF–PN admixtures at the volume ratio 1:1 precipitation was observed in formulations with lower concentrations of magnesium and calcium ions in which the molar ratio of magnesium to calcium ions was 2:1. This may be associated with the formation of a magnesium adduct with water molecules, which results in reduced CF solubility and precipitation of CF zwitterions. To confirm the identity of CF in precipitate, we performed FT-IR analysis. The measured spectrum of precipitate presented similar absorption bands to the spectrum of the reference sample and CF spectrum showed in the literature [28]. The spectra were characterized by the high matching coefficient, expressed as a purity factor equal to 0.9552. The spectrum of precipitate presented the characteristic bands for CF in the fingerprint region. The sharp peak at 1724.37 cm^−1^ observed in the precipitate and less formed in the reference sample could be due to the disruptive interference of the solvent.

**Figure 3:**
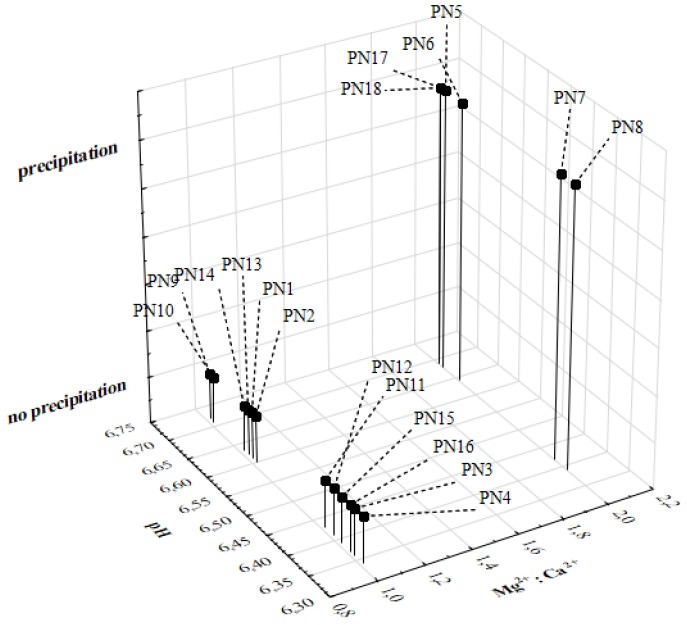
Relationships between the pH of the PN admixtures, the molar ratio of magnesium and calcium ions, and observations concerning precipitation.

Regarding the CF–PN admixtures for which no precipitation was observed, the molar ratio of magnesium to calcium ions was 1:1 (*v*/*v*), which was probably associated with a greater affinity of calcium ions for CF molecules and the formation of well-soluble complexes, more hydrophilic than CF. Figure 3 presents relationships between the pH of the PN admixtures, the molar ratio of magnesium and calcium ions, and observations concerning precipitation.

In this study, the presence or absence of precipitation did not correlate with the physical parameters of the CF–PN admixtures, which did not exceed the pharmacopeial value (MDD > 500 nm) [22]. It should be noted that a sole reliance on the physical parameters of CF–PN admixtures in clinical practice may endanger the patient’s health by administering a drug of inadequate quality.

The MDD of the lipid emulsion did not increase in the CF–PN admixtures for which precipitation was observed. This was due to the sedimentation of the precipitate to the bottom of the measuring cuvette, which did not interfere with the measurement of this parameter. The ZetaSizer Nano ZS used in the tests to measure the distribution of particle sizes for stable emulsions and suspensions ranging in size from 0.3 nm to 10 µm. In this method, the dispersion of nanoparticles is illuminated by a laser beam. The laser light is scattered and next captured by the detector. The speed of particles is measured on the basis of Brownian motion and then converted to particle-size distribution, using the Stokes–Einstein equation. Therefore, in the case of precipitation of insoluble particles, visual and/or microscopic assessment is necessary, as the measurement of the particle-size distribution alone can cause false positive results.

The lack of compatibility between CF and PN admixtures containing only lipid emulsion and glucose was observed by Omotani et al. [29] immediately after sample preparation. The authors mixed a 5% glucose solution with a 20% Intralipos fat emulsion (100% soybean oil) with CF at a volume ratio of 33:10:40, and then counted the number of large insoluble particles by using the light obscuration method upon sample preparation and after 1, 3, 6, 12, and 24 h of storage. The results indicated particles with a diameter ranging from 25 to 50 μm directly after sample preparation. The occurrence of above 0.05% particles exceeding 5 µm in diameter disqualifies the CF–PN admixture from administration [22].

## 5. Limitations

The aim of our study was to evaluate the compatibility of CF with selected PN admixtures. Determining the mechanism of observed interaction would be very interesting but also very difficult to implement. It should be emphasized that the PN admixture is one of the most complex drugs used in modern medicine and pharmacy. By analyzing its composition, more than 30 substances can be detected that jointly affect the drug combined with the PN admixture. Explanation of the mechanism of formation of CF precipitation would require the preparation of another several dozen model PN admixtures with increasing ion concentrations, their different molar ratios, and different contents of other components of the PN admixture affecting such parameters as even pH. It was also not advisable to prepare simpler systems, e.g., without a lipid emulsion or amino acid solution, because each of these components may indirectly affect the solubility of CF, e.g., by changing the pH. That is why we determined the compatibility of CF with selected PN admixtures used in clinical practice. Our hypothesis concerning the impact of pH and molar ratios of selected ions was an attempt to find factors responsible for the interaction and was not in itself an explanation of the mechanism of observed interactions.

## 6. Conclusions

The compatibility tests showed that the addition of the CF to the PN admixtures did not cause any color change or sign of destabilization in the fat emulsion. However, precipitation was observed in formulations containing higher CF concentrations and, in the case of lower CF concentrations, in formulations containing magnesium and calcium ions at a molar ratio of 2:1. Due to the inconclusive findings regarding compatibility between the CF and the PN admixtures, it is advisable that the administration of CF–PN admixtures via the Y-site should be avoided or performed only with PN admixtures for which compatibility has been confirmed and the CF concentration in samples is 1 mg/mL at a molar ratio of magnesium to calcium ions of 1:1.

## Figures and Tables

**Table 1:** Composition and characteristic of PN admixtures.

Ingredient/Parameter	Unit	PN1	PN2	PN3	PN4	PN5	PN6	PN7	PN8	PN9	PN10	PN11	PN12	PN13	PN14	PN15	PN16	PN17	PN18
Amino Acid	g	27	27	63	63	27	27	63	63	27	27	63	63	27	27	63	63	27	27
Glucose		180	180	180	180	180	180	180	180	180	180	180	180	180	180	180	180	180	180
Lipid Emulsion		9 ^a^	45 ^a^	9 ^a^	45 ^a^	9 ^a^	45 ^a^	9 ^a^	45 ^a^	9 ^a^	45 ^a^	9 ^a^	45 ^a^	9 ^b^	45 ^b^	9 ^b^	45 ^b^	45 ^b^	9 ^b^
Water		893	713	549	369	928	748	583	403	966	789	611	434	894	714	549	369	748	928
Sodium	mmol	117	117	117	117	117	117	117	117	72	66	82	77	117	117	117	117	117	117
Potassium		99	99	99	99	99	99	99	99	7	7	16	16	99	99	99	99	99	99
Magnesium		7	7	7	7	2	2	2	2	7	7	7	7	7	7	7	7	2	2
Calcium		7	7	7	7	1	1	1	1	7	7	7	7	7	7	7	7	1	1
Phosphate		56	56	89	89	56	56	56	56	89	89	56	56	56	56	89	89	56	56
Total Energy	kcal	909	1233	1053	1377	909	1233	2021	1697	2567	2248	2072	1753	900	1233	1233	1377	1233	909
Theoretical Osmolarity	mOsm/L	919	959	1093	1134	919	959	1094	1134	757	791	956	989	918	956	1093	1131	956	918
Total Volume	mL	1800	1800	1800	1800	1800	1800	1800	1800	1800	1800	1800	1800	1800	1800	1800	1800	1800	1800

a = Lipofundin MCT/LCT 20%; b = Smoflipid 20%.

**Table 2:** Results of pH, osmolality, zeta potential, and particle size of PN admixtures.

PN Admixture	pH ± SD		Osmolality ± SD (mOsm/kg H_2_O)		Zeta Potential ± SD (mV)		MDD ± SD (nm)	
	0 h	4 h	0 h	4 h	0 h	4 h	0 h	4 h
PN1	6.61 ± 0.01	6.60 ± 0.00	996 ± 5	997 ± 3	−11.12 ± 1.09	−11.53 ± 0.85	219.7 ± 1.9	215.7 ± 2.9
PN2	6.60 ± 0.00	6.61 ± 0.01	1076 ± 4	1076 ± 5	−11.40 ± 1.00	−11.33 ± 0.85	220.0 ± 1.4	218.3 ± 1.5
PN3	6.36 ± 0.00	6.37 ± 0.01	1217 ± 3	1217 ± 2	−9.19 ± 0.43	−9.89 ± 0.09	219.2 ± 1.1	218.7 ± 1.9
PN4	6.34 ± 0.01	6.34 ± 0.01	1281 ± 3	1281 ± 5	−8.38 ± 0.15	−7.88 ± 0.58	220.6 ± 1.6	221.7 ± 0.9
PN5	6.68 ± 0.00	6.69 ± 0.01	992 ± 3	992 ± 4	−17.50 ± 1.05	−14.73 ± 1.46	216.5 ± 2.4	217.0 ± 3.5
PN6	6.64 ± 0.00	6.65 ± 0.01	1057 ± 6	1057 ± 7	−16.37 ± 0.75	−15.77 ± 0.32	219.4 ± 2.6	220.8 ± 2.1
PN7	6.42 ± 0.01	6.40 ± 0.01	1172 ± 4	1172 ± 5	−11.58 ± 2.92	−10.30 ± 2.51	222.0 ± 1.5	223.9 ± 3.7
PN8	6.39 ± 0.01	6.38 ± 0.01	1278 ± 3	1278 ± 4	−13.87 ± 2.46	−13.87 ± 1.85	224.7 ± 3.6	221.3 ± 2.5
PN9	6.72 ± 0.01	6.73 ± 0.01	848 ± 1	848 ± 3	−13.00 ± 0.10	−13.50 ± 1.13	221.0 ± 1.0	216.8 ± 2.0
PN10	6.71 ± 0.01	6.70 ± 0.00	900 ± 5	899 ± 5	−12.87 ± 1.90	−14.67 ± 0.67	223.4 ± 0.4	221.9 ± 0.9
PN11	6.43 ± 0.01	6.42 ± 0.01	1042 ± 1	1042 ± 3	−9.83 ± 0.99	−7.42 ± 1.88	220.4 ± 1.4	219.3 ± 0.9
PN12	6.41 ± 0.01	6.40 ± 0.00	1136 ± 7	1135 ± 5	−10.00 ± 0.78	−9.91 ± 0.25	221.8 ± 2.1	219.6 ± 0.8
PN13	6.62 ± 0.01	6.61 ± 0.01	994 ± 3	993 ± 3	−17.97 ± 0.81	−19.17 ± 2.31	336.7 ± 3.6	334.5 ± 4.4
PN14	6.63 ± 0.00	6.63 ± 0.00	1053 ± 3	1053 ± 2	−18.37 ± 3.26	−16.80 ± 1.15	335.0 ± 1.9	329.5 ± 4.4
PN15	6.39 ± 0.01	6.39 ± 0.00	1197 ± 2	1196 ± 6	−12.70 ± 0.60	−13.10 ± 0.61	338.8 ± 3.4	334.8 ± 3.6
PN16	6.37 ± 0.01	6.38 ± 0.01	1271 ± 3	1271 ± 2	−11.27 ± 0.31	−13.00 ± 0.89	330.9 ± 4.9	330.4 ± 3.1
PN17	6.69 ± 0.01	6.69 ± 0.00	1047 ± 3	1048 ± 5	−15.83 ± 1.10	−18.33 ± 1.12	329.0 ± 2.8	326.1 ± 5.5
PN18	6.69 ± 0.00	6.69 ± 0.01	979 ± 4	978 ± 6	−22.47 ± 0.81	−23.80 ± 1.31	328.6 ± 3.2	335.0 ± 6.4

**Table 3:** Results of pH, osmolality, zeta potential, and particle size of CF–PN samples.

PN Admixture	CF:PN Volume Ratio	pH ± SD		Osmolality ± SD (mOsm/kg H_2_O)		Zeta Potential ± SD (mV)		MDD ± SD (nm)	
		0 h	4 h	0 h	4 h	0 h	4 h	0 h	4 h
PN1	1:1	6.24 ± 0.01	6.24 ± 0.01	607 ± 3	601 ± 5	−7.26 ± 0.47	−8.22 ± 0.35	212.9 ± 2.8	212.7 ± 2.7
PN1	2:1	6.04 ± 0.01	6.04 ± 0.00	494 ± 3	495 ± 3	−5.72 ± 0.73	−5.54 ± 0.23	218.7 ± 2.3	210.0 ± 3.3 *
PN2	1:1	6.22 ± 0.01	6.22 ± 0.00	633 ± 3	629 ± 3	−6.49 ± 0.20	−7.07 ± 0.61	216.4 ± 2.7	214.0 ± 1.4
PN2	2:1	6.03 ± 0.01	6.03 ± 0.01	511 ± 2	510 ± 1	−3.63 ± 0.51	−4.43 ± 0.09	213.4 ± 0.8	212.4 ± 2.7
PN3	1:1	6.14 ± 0.00	6.14 ± 0.00	701 ± 2	712 ± 3	−5.87 ± 0.16	−7.04 ± 0.42	215.0 ± 2.5	212.8 ± 1.7
PN3	2:1	6.00 ± 0.01	6.00 ± 0.01	548 ± 7	545 ± 3	−3.31 ± 0.27	−3.41 ± 0.31	214.3 ± 2.5	212.4 ± 1.7
PN4	1:1	6.09 ± 0.01	6.09 ± 0.01	724 ± 2	725 ± 1	−4.86 ± 0.31	−5.41 ± 0.51	214.5 ± 3.7	214.4 ± 2.0
PN4	2:1	6.00 ± 0.01	5.93 ± 0.02 *	554 ± 4	555 ± 3	−2.50 ± 0.15	−2.44 ± 0.54	215.3 ± 0.6	212.5 ± 2.2
PN5	1:1	6.32 ± 0.01	6.31 ± 0.01	601 ± 1	608 ± 1	−9.02 ± 0.80	−10.26 ± 0.33	214.3 ± 0.9	211.0 ± 0.6
PN5	2:1	6.15 ± 0.01	5.95 ± 0.01 *	493 ± 6	492 ± 3	−4.91 ± 0.60	−7.27 ± 0.09	215.6 ± 2.8	209.7 ± 1.6
PN6	1:1	6.29 ± 0.01	6.29 ± 0.01	634 ± 4	638 ± 5	−8.46 ± 0.48	−10.31 ± 0.55	214.6 ± 2.3	213.2 ± 3.0
PN6	2:1	6.12 ± 0.01	5.98 ± 0.01 *	511 ± 6	511 ± 3	−4.72 ± 0.63	−6.10 ± 0.19	215.2 ± 2.3	210.2 ± 0.8
PN7	1:1	6.21 ± 0.01	6.12 ± 0.00 *	689 ± 4	686 ± 1	−8.91 ± 0.62	−12.80 ± 0.56 *	216.3 ± 2.2	214.4 ± 2.4
PN7	2:1	6.08 ± 0.00	5.88 ± 0.01 *	540 ± 2	547 ± 5	−7.63 ± 0.23	−10.77 ± 0.32	214.5 ± 2.1	212.4 ± 2.4
PN8	1:1	6.17 ± 0.01	6.10 ± 0.00 *	730 ± 5	715 ± 2 *	−7.93 ± 0.62	−9.20 ± 0.41	218.6 ± 3.1	216.1 ± 4.3
PN8	2:1	6.04 ± 0.02	5.87 ± 0.01 *	558 ± 1	564 ± 1	−5.26 ± 0.31	−6.01 ± 0.23	216.6 ± 4.1	214.2 ± 4.0
PN9	1:1	6.26 ± 0.01	6.28 ± 0.01	535 ± 5	531 ± 8	−10.00 ± 0.36	−12.37 ± 0.32	215.8 ± 2.9	211.7 ± 3.6
PN9	2:1	6.06 ± 0.01	6.04 ± 0.01	441 ± 6	441 ± 5	−6.66 ± 0.96	−8.52 ± 0.91	214.5 ± 3.7	214.9 ± 2.7
PN10	1:1	6.23 ± 0.01	6.24 ± 0.01	553 ± 1	555 ± 5	−8.72 ± 0.23	−8.77 ± 0.63	217.7 ± 4.6	214.2 ± 3.1
PN10	2:1	6.05 ± 0.01	6.00 ± 0.02 *	453 ± 5	455 ± 5	−5.41 ± 0.67	−5.68 ± 0.52	215.0 ± 4.4	214.7 ± 4.3
PN11	1:1	6.06 ± 0.01	6.06 ± 0.01	634 ± 0	626 ± 3	−7.33 ± 0.26	−7.46 ± 0.72	220.6 ± 4.0	216.1 ± 1.6
PN11	2:1	5.90 ± 0.00	5.90 ± 0.01	503 ± 7	502 ± 6	−6.22 ± 0.43	−5.82 ± 0.35	212.5 ± 2.2	211.9 ± 1.9
PN12	1:1	6.13 ± 0.01	6.15 ± 0.02	654 ± 4	653 ± 5	−7.13 ± 0.47	−6.81 ± 0.19	218.4 ± 4.7	213.1 ± 3.1
PN12	2:1	6.03 ± 0.01	5.88 ± 0.02 *	521 ± 4	520 ± 3	−4.79 ± 0.54	−5.32 ± 0.14	214.5 ± 3.7	214.7 ± 3.3
PN13	1:1	6.29 ± 0.01	6.25 ± 0.02	613 ± 4	610 ± 5	−9.93 ± 0.70	−15.80 ± 0.40 *	323.8 ± 2.7	323.6 ± 3.6
PN13	2:1	6.08 ± 0.01	6.04 ± 0.00	497 ± 7	497 ± 2	−14.43 ± 0.49	−14.13 ± 1.63	325.7 ± 7.0	330.0 ± 8.3
PN14	1:1	6.26 ± 0.01	6.26 ± 0.01	628 ± 1	629 ± 4	–12.97 ± 0.55	−11.27 ± 1.21	326.5 ± 6.4	326.7 ± 5.2
PN14	2:1	6.06 ± 0.01	6.05 ± 0.01	503 ± 1	506 ± 3	–10.47 ± 1.14	−11.57 ± 1.31	322.4 ± 7.0	317.9 ± 1.7
PN15	1:1	6.13 ± 0.01	6.12 ± 0.00	681 ± 3	675 ± 3	−11.47 ± 0.85	−11.13 ± 0.76	323.9 ± 2.2	319.9 ± 5.2
PN15	2:1	5.99 ± 0.01	5.99 ± 0.01	537 ± 3	533 ± 3	−11.30 ± 0.30	−9.10 ± 0.93	325.5 ± 5.0	320.5 ± 5.3
PN16	1:1	6.13 ± 0.01	6.14 ± 0.00	723 ± 6	717 ± 2	−10.45 ± 0.70	−8.85 ± 0.91	323.4 ± 5.4	324.3 ± 1.1
PN16	2:1	5.99 ± 0.01	6.00 ± 0.02	563 ± 4	559 ± 1	−11.17 ± 0.75	−6.75 ± 1.25 *	321.8 ± 4.8	324.7 ± 3.5
PN17	1:1	6.33 ± 0.02	6.27 ± 0.01 *	624 ± 1	626 ± 5	−16.27 ± 0.71	−14.63 ± 0.55	329.0 ± 10.6	325.2 ± 4.5
PN17	2:1	6.17 ± 0.02	5.89 ± 0.01 *	501 ± 6	503 ± 3	−15.27 ± 1.02	−11.83 ± 0.75	328.4 ± 1.7	328.5 ± 4.3
PN18	1:1	6.32 ± 0.02	6.22 ± 0.02 *	602 ± 5	597 ± 8	−26.30 ± 0.56	−18.13 ± 0.40 *	326.4 ± 4.1	332.7 ± 7.2
PN18	2:1	6.14 ± 0.01	5.82 ± 0.02 *	484 ± 1	484 ± 5	−21.03 ± 2.06	−12.53 ± 0.7 *	325.8 ± 6.0	329.5 ± 8.3

* = significance *p* < 0.05 differences between value of *t* = 4 h and *t* = 0 h.

**Table 4:** Characteristic bands of precipitate and reference sample in FT-IR spectra.

Precipitate	Reference Sample	Peak Assignment
Wavenumbers (cm^−1^)		
3436.21	3444.15	–OH stretching vibration
1621.67	1616.36	C=O stretching vibration
1587.91	1592.21	C=C stretching vibration
1284.60	1282.67	C–N stretching vibration
1036.26	1035.78	C–F stretching vibration
722.34	722.34	–NH vibration
